# Therapeutic Applications of Biogenic Silver Nanomaterial Synthesized from the Paper Flower of *Bougainvillea glabra* (Miami, Pink)

**DOI:** 10.3390/nano13030615

**Published:** 2023-02-03

**Authors:** Mohammad Oves, Mohd Ahmar Rauf, Huda A. Qari

**Affiliations:** 1 Centre of Excellence in Environmental Studies, King Abdulaziz University, Jeddah 22252, Saudi Arabia; 2 Miller School of Medicine, University of Miami, Miami, FL 33136, USA; 3 Biological Science Department, Faculty of Science, King Abdulaziz University, Jeddah 22252, Saudi Arabia

**Keywords:** biogenic, biocompatible, AgNPs, *Bougainvillea glabra*, antibacterial, anticancer

## Abstract

In this research, *Bougainvillea glabra* paper flower extract was used to quickly synthesize biogenic silver nanoparticles (BAgNPs) utilizing green chemistry. Using the flower extract as a biological reducing agent, silver nanoparticles were generated by the conversion of Ag^+^ cations to Ag^0^ ions. Data patterns obtained from physical techniques for characterizing BAgNPs, employing UV-visible, scattering electron microscope (SEM), transmission electron microscope (TEM), dynamic light scattering (DLS), X-ray diffraction (XRD), and Fourier-transform infrared spectroscopy (FTIR), suggested that the nanoparticles have a spherical to oval form with size ranging from 10 to 50 nm. Spectroscopy and microscopic analysis were used to learn more about the antibacterial properties of the biologically produced BAgNPs from *Bougainvillea glabra*. Further, the potential mechanism of action of nanoparticles was investigated by studying their interactions in vitro with several bacterial strains and mammalian cancer cell systems. Finally, we can conclude that BAgNPs can be functionalized to dramatically inhibit bacterial growth and the growth of cancer cells in culture conditions, suggesting that biologically produced nanomaterials will provide new opportunities for a wide range of biomedical applications in the near future.

## 1. Introduction

The synthesis of silver nanoparticles from plant sources is always preferable from an ecological perspective. Biogenic silver nanoparticles (BAgNPs) are metallic colloidal particle sizes that range between 1 and 100 nm. Metallic nanomaterials have recently gained a lot of attention for their mechanical, barrier, thermal, optical, and biological properties. These inherent properties of BAgNPs increase their usefulness in catalysis, electrochemistry, biomedicines, pharmaceuticals, sensors, and cosmetics, for the production of antibacterial drugs, wound healing materials, drug delivery vehicles, and nanocomposite films in food packages. Previously, various chemical processes were employed to suit the needs of the medical and non-medical industries for AgNPs discovered through study.

Even now, the development of cost-effective, ecologically safe technologies for producing nanoparticles is a fundamental theme of nanotechnology. However, by using plants or microbial sources as reducing agents, AgNPs with little or no toxicity can be produced. Silver nanoparticles are attractive nanomaterials due to their biocompatibility and antibacterial properties [1]. Moreover, because of their biological properties, they are a desirable, active component for the development of medicines, drug delivery vehicles, diagnostics, and treatments [2,3,4]. Therefore, the synthesis of AgNPs is an arduous process. Chemical reduction and autoclaving, gamma radiation, and electrochemical processes have previously produced high yields of AgNPs, but they were energy-intensive and generated harmful byproducts [5]. The toxic chemicals and the capping and reducing agents have detrimental effects on the environment. In order to synthesize biomedical AgNPs, plant extracts are used. The use of biological agents is biocompatible, biodegradable, environmentally friendly, and cost-effective, and they limit particle agglomeration and minimize metal precursors [6]. The nanomaterials created by using green synthesis techniques and a variety of plants have greater antibacterial properties than those that are produced by thermal or chemical means. As a result of their unique chemical properties, the nanoparticles created from aqueous floral extract are more effective than those obtained from other biological sources [7]. The flower extract contains secondary compounds that assist as precursor molecules as reducing and stabilizing agents for nanomaterials’ formation. Medicinal plant flower extracts are employed as non-hazardous reducing agents in the biogenic production of AgNPs [8]. AgNPs produced by plants were previously studied, and intriguing facts were revealed. In the synthesis of NPs, jasmine extract has been found to serve as a silver ion-reducing agent and nanomaterial stabilizing agent [9]. AgNPs produced by flowers possess antibacterial, antioxidant, and insecticidal properties that can be used in a variety of applications. A variety of silver, zinc, and gold nanoparticles derived from *Rhanterium epapposum* flower extract have been found to be effective cytotoxic and antifungal agents [10]. There have been no published studies examining the production of silver nanoparticles from *Bougainvillea glabra*, which belongs to the Malvaceae family.

Around the world, infectious diseases caused on by harmful microorganisms affect the lives of millions of people and are responsible for a considerable number of deaths. Antibiotic abuse and misuse result in drug resistance in various bacteria, rendering treatments ineffective against the disease-causing agent. Every year, the number of *Escherichia coli* outbreaks increases, leading to hemolytic uremia syndrome [11]. Sometimes, consuming contaminated food with *E. coli* causes gastroenteritis, developing into a fever, meningitis, sepsis, and urinary tract infection. However, *Staphylococcus aureus* is a pathogenic bacterium, causing invasive infections, sepsis, and death. In consonance with the Centers for Disease Control and Prevention, bloodstream infections caused by *Staphylococcus aureus* (staph) infected more than 119,000 Americans in 2017, with 20,000 deaths [12,13]. *S. aureus* can cause infection in the skin and soft tissue, osteoarticular, endocarditis infections, sepsis, pneumonia, and several other infections [14,15]. The pathogen’s ability to synthesize various enzymes and toxins, particularly positive coagulase, is essential because it is frequently linked to the humans’ etiology of many infections and illnesses [16,17]. Despite advances in avoiding methicillin-resistant *S. aureus* (MRSA) pathogen spreading in clinical surroundings, a critical evaluation of the difficulty in healthcare and social sites is required [18]. With new antibacterial agents, efficient control of these pathogenic bacteria is thus highly desirable. Biosynthesis of nanomaterials draws on nature’s extensive repertoire of strategies for the synthesis of nanoscale and microscale inorganic materials. Green chemistry concepts are consistent with bio-organic synthesis [19,20]. Environmentally friendly, nontoxic, and safe chemicals are used in the “green synthesis” of nanoparticles. Biotechnologically or environmentally produced nanoparticles have several desirable properties, including high stability and size. Many different methods, including chemical, physical, and biological processes, can be used to generate nanoparticles [21,22,23,24,25,26]. There is a risk to human and environmental health from the employment of chemicals and physical procedures due to the high levels of radiation, highly specific reductants, and potentially hazardous agents. Due to this, the biological synthesis of nanoparticles has the potential to be a one-step eco-friendly bio-reduction approach that requires significantly less energy than traditional synthesis. *Bougainvillea galabra* extracts contain a number of phytochemicals, such as pinitol, betacyanine, flavonoids, tannins, and alkaloids as some of the identified active components [27] that are effective in reducing environments for metal ions, such as ZnO and CuO, to be reduced into nanoparticles [28,29]. In addition to phytol, methyl 2-hydroxybenzoate, pinitol, terpinines-(E)-ionone, and linalool, Bougainvillea phytochemicals may also provide a favorable environment for nanoscale biomineralization. Additionally, it has been hypothesized that the leaves offer anti-inflammatory, antimicrobial, and anthelmintic properties [30], which prompted us to employ *Bougainvillea galabra* extract to develop biogenic AgNPs, that are useful in biomedical applications. It was found that these green AgNPs had outstanding anticancer activity regarding lung cancer cells as well as antibacterial activity against pathogenic bacteria.

## 2. Materials and Methods

### 2.1. Chemicals and Components

A pure high-grade silver nitrate, DCFDA, Hoechst 33342, Acridine orange, and EtBr were bought from Sigma–Aldrich Co. (St. Louis, MO, USA). Agarose low EEO and all routine reagents were obtained from Merck Pvt. Ltd. The cancer cell specific for human lung cancer cells (A549) was purchased from ATCC, USA. Culture medium for bacteria growth was acquired from HiMedia (Pvt. Ltd., Mumbai, India).

### 2.2. Synthesis and Characterization of BAgNPs

Blooms of Bougainvillaea glabara, also known as Miami Pink, were sourced from a street garden in Miami, in the United States, as well as the botanical garden at King Abdulaziz University in Jeddah, Saudi Arabia, for the purpose of producing biogenic silver nanoparticles. Here, 200 g of blooms was initially cleaned three times with filtered water and dried in the shade. The outer green cover of the dried flower was removed, and an estimated 10 g of flower was used to make a powder with the grinder. This powder was amended into a 250 mL capacity flask and dissolved with 100 mL of distilled water and positioned in the oven for two hours at 80 °C. After incubation, this plant extract was decanted and filtered with Whatman paper, and then was placed in a refrigerator at 4 °C for future use. The flower extract from 1 to 5 mL was gradually amended into 50 mL of a 1 mM AgNO_3_ liquid solution. This reaction mixture was watched over time and a color change from light yellow to brown was noticed in a solution that amply demonstrated the involvement of the biogenic material, with the reduction of Ag^+^ into metallic silver Ag^0^ as nanoparticles, as measured by a UV-visible spectrophotometer. The final reaction sample was collected and centrifuged for 15 min at a speed of 25,000× *g* to create a pellet that could be used in further experiments. In this technique, the paper flower extract serves as a reducing and stabilizing/capping agent to fabricate the nanoparticles. This bottom-up technique for nanoparticle synthesis is very simple, practical, effective, economical, and environmentally benign. This method does not require a chemical catalyst, a vacuum, energy, or an extreme temperature at any step.

#### 2.2.1. Particle Characterization

The biogenic BAgNPs were amended into the DMEM medium containing 10% FBS and placed with a sonicator probe for 10 min at 30 watts (Sonics & Material Inc., 53 Church Hill Rd, Newtown, CT, 06470, United States America). A Zeta-sizer Nano-ZS, fitted with a 4.0 mW laser, at 633 nm, for dynamic light scattering was used in this study (Malvern Ltd., Malvern, UK, Model ZEN3600). In this technique, dynamic light scattering was employed to estimate the size and zeta potential of the material. Further, the sample was prepared for transmission electron microscopy (TEM), which was performed through the drop-coating method, where the 10 µg/mL sample solution of AgNPs was placed onto grids and it was previously covered with carbon tape. Prior to measurement, the film as a coating on the TEM grids was allowed time to dry. The Tecnai TM G2 Spirit apparatus was used for the material imaging experiments and was run at an accelerated voltage of 80 kV (TEM, FEI, Amsterdam, The Netherlands).

#### 2.2.2. UV-Visible Spectroscopy

The optimization of the Ag^+^ reduction using the paper flower of Miami pink *Bougainvillea glabara* extract as a substrate in combination of silver nitrate solution able to produced a colloidal mixture of AgNPs. The obtained colloidal solution was analyzed by the UV-vis absorption, with the obtained spectra serving as a function of both time and concentration (0.1–1.5 mL). The standard quartz 10 mm cuvette was used during the investigation (Shimadzu UV/Vis spectrophotometer, optical windows: 2, spectral range: 190–2500 nm, pathlength: 10 nm, volume: 3.5 mL). In the process of tracking the bio-reduction of Ag^+^ in an aqueous solution that is being examined, approximately 10 milliliters of colloidal samples are typically used. Incubation durations of 10, 30, 60, and 120 min were used for the tests involving the UV-vis spectrum. Furthermore, reactions at variable temperatures of 6, 12, 25, 50, 75, 100, and 150 °C, and pH 3, 4, 5, 6, 7, 8, 9, and 10, were investigated for nanoparticle synthesis optimization and reported in the Supplementary File (Figures S1 and S2), [8,31,32].

#### 2.2.3. Fourier-Transform Infrared (FTIR) and X-ray Diffraction (XRD)

Fourier transformations have also been used to investigate infrared (Shimadzu, Kyoto, Japan) spectra for biogenic nanomaterials that have been produced. In order to determine the location of the biological entities that are engaged in the production of particles, the biogenic AgNPs with KBr crystal were used as a beam splitter. After centrifuging the sample for ten minutes at a speed of ten thousand revolutions per minute, the resultant pellets were dried at a temperature of 80 °C and crushed to eliminate any undesired plant materials and silver that included KBr crystal. The machine range of 400–4000 cm^−1^ was set for the spectra of AgNPs to be recorded.

A crystal study of fine AgNPs was conducted via X-ray diffraction patterns. XRD was accomplished with Cu-Kα radiation (l = 0.15406 nm) using an X-pert X-ray powder diffractometer (Philips PW1398, Materials Research Laboratory, UC Santa Barbara, Isla Vista, CA 93106, USA) in the 2 θ range of 20 to 80° [8,31,32].

### 2.3. Purification and Release Kinetics of BAgNPs

The Miami Pink flower of *Bougainvillaea glabra* extract was used to create the biogenic AgNPs. To remove the residual silver ions and plant extract, the produced nanomaterials were cleansed four times with Milli Q ultrapure water, dried, and dispersed. After that, the components were transferred to the HEPES solution and put in the dialysis bag. Further, the material-containing bag was re-suspended in 10 mL of HEPES buffer solution (20 mM) at pH 7.4 and a sucrose mixture was added to create a 2.5 g/mL density. Before being collected for additional analysis, the nanoparticle suspension was centrifuged at 10,000 rpm at 8 °C for 30 min, with 10 mL of a linear gradient of sucrose density (0.25–1.0 M). The amount of nanoparticles in the reaction mixture was assessed using ICP-AES (Shimadzu, Japan). The biogenic AgNPs were put in a dialysis bag with a 12 kDa cutoff pore size range. By suspending these bags in a buffer solution (100 mL of HEPES at pH 7.4) for an extended period, the release percentage and order of reaction kinetics of nanomaterials in a liquid medium were investigated. The amount of released ions was recorded using ICP-AES. A 1 mL colloidal solution of AgNPs was combined with 3 mL of sulfuric acid and 9 mL of concentrated nitric acid to perform the digestion that breaks down AgNPs into their atomic form. For total salvation, the reaction sample was also heated on a hot plate for 1 h to 110 °C. The primed sample was examined in the occurrence of an air/acetylene flame with a 1.0 to 1.2 L/min flow rate and atomized at 1200 °C temperature. Further, the calculation of the percentage of silver ions released was performed according to the following equation:

$$\mathrm{Silver}\;\mathrm{ion}\;\mathrm{released}\;(\%)\;=\left[\frac{D_{x}}{D_{i}}\right]\times 100$$

where D_i_ represents the total Ag in the dialysis bag at the time, and D_x_ is the released ions in the buffer solution.

### 2.4. Antibacterial Properties of As-Synthesized AgNPs

The antimicrobial nature of the biogenic AgNPs was studied following the NCCLS mandate. Detailed information on the methodology is discussed below.

#### 2.4.1. Bacterial Strains and Cell Culture Conditions

For antibacterial studies, different ATCC and clinical isolates of bacteria were obtained from King Abdulaziz University Hospital Microbiology Lab, King Abdulaziz University Jeddah, Saudi Arabia. *E. coli*, as a Gram-negative, and *S. aureus* as a Gram-positive bacteria, were selected as the target test organisms. Luria–Bertani (LB) medium and nutrient agar were used to cultivate bacteria at 37 °C on a rotary platform of the incubator chamber. The optical density (OD) of liquid culture was measured at 600 nm wavelength filter, which reflects the bacterial cells in the liquid broth.

#### 2.4.2. Zone Inhibition Assay

Testing on bacteria revealed that the biogenic nanoparticles showed antibacterial characteristics. *E. coli* and *Staphylococcus aureus* were used in this study’s antibacterial testing. The university culture bank provided these microbial cultures. The Bauer’s diffusion method was used to conduct development inhibition experiments in both liquid and solid agar plate media. Bacteria were cultured and transferred using McFarland’s vial culture method in a sterile medium. The McFarland protocol is the main source used to evaluate the number of bacteria in the samples. Bacteria were seeded on nutrient agar to promote the growth of these microorganisms. The 6 mm bore puncture was used to create a well in the media plate, and afterward, the lower side of these wells was sealed with molten agar solution. Variable AgNP samples were added to the wells of the media plate before being incubated overnight at 35 °C [8,31,32].

#### 2.4.3. Anti-Biofilm Activity

The biofilm test was conducted using the XTT methodology. Briefly, the PBS was used to erode the fresh biofilm growth in the plate (96 wells) and eliminate the unattached bacterial cells in the sample. The biofilms were developed and then treated for 48 h with different doses of AgNPs and conventional antibiotics. Additionally, crystal violet (CV) staining was performed to evaluate biofilm growth. The biofilm-formed culture wells were eroded multiple times with PBS buffer, and a paper towel was used to remove moisture from the inverted plate. In addition, a 0.1% crystal violet solution was applied to the dry wells and incubated at 25 °C for 15 min to determine the biofilm thickness [8,31,32,33].

### 2.5. Cytotoxicity Assay

#### Erythrocyte Lysis In Vitro Assay

The RBC lysis in vitro test was accomplished to study the toxicity of biogenic AgNPs by evaluating the released hemoglobin. The detailed methodology of the RBC lysis assay is described in the Supplementary Material (S.M.) file.

### 2.6. Anticancer Activity of As-Synthesized AgNPs

To study the anticancer activities of BAgNPs, lung cancer cells (A549) were employed. The cells were cultivated in DMEM media with FBS (10%) in CO_2_ (5%), relative humidity 95%, in the chamber at 37 °C. After incubation, the cells were harvested at 90% confluency, eroded with saline buffer, and then trypsinized with trypsin (0.25%) and EDTA (0.03%) solution. Successively, the cells were transferred to the 96-well culture plate and endorsed to incubate at 37 °C for 24 h. To assess the cell viability of the AgNPs, the MTT assay was performed [31].

#### 2.6.1. BAgNPs Induced Reactive Oxygen Species (ROS) Production in the Cancer Cells

The ROS production assay was accomplished to elucidate the mechanism of cancerous cell toxicity induced by BAgNPs and oxidative stress. The reactive oxygen species (ROS) production was determined by staining cells with DCFH dye, a cell-permeable dye, considering that the fluorescence intensity is relative to the extent of ROS produced. Concisely, 2 × 10^4^ A549 cells were treated with 25 and 50 μg/mL concentrations for 24 h in 2 sets. Afterward, the cells were subjected to 20 µM of DCFH-DA for incubation at 37 °C for 30 min. The treated cells were harvested, eroded with ice-cold PBS, and examined by a confocal fluorescence microscope (Zeiss, Oberkochen, Germany) [31].

#### 2.6.2. Mitochondrial Potential

Rhodamine 231 was used in this investigation to assess the mitochondrial potential (BD BIO, Franklin Lakes, NJ, USA). The cells were exposed to diverse concentrations of AgNPs, and subsequently, cells were fixed with 4% paraformaldehyde and kept for 30 min at 25 °C. The detailed process has been described in the S. M. file. Subsequently, after washing with PBS, the cells were stained with Rhodamine 231 dye. After rinsing the cells with PBS, their fluorescent intensity was studied under fluorescence microscopy at 10× magnification [8,31].

#### 2.6.3. Apoptosis Analyzed by Hoechst Dye

Hoechst 33342 was used in this investigation to assess apoptotic nuclear morphology (BD BIO, St Louis, MO, USA). The cultured cells were exposed to different concentrations of AgNPs, and subsequently, cells were fixed with 4% paraformaldehyde and kept for 30 min at 25 °C. After washing with PBS, the cells were stained with Hoechst 33342 (2 g/mL) and raised to 37 °C for 30 min. After rinsing the cells with distilled water, their morphology was studied under fluorescence microscopy at 10× magnification [8,31].

#### 2.6.4. Biogenic Nanomaterials’ Antioxidant Activity

The antioxidant property of biogenic silver nanomaterials has been tested according to the standard protocol. A series of solutions from the stock solution of colloidal silver nanomaterials taken up to 1 mL, and a 1 mL solution of 1,1-diphenyl-2-picrylhydrazulin (from the stock solution of 2.5 μg/mL of 1 DPPH) were actively mixed and raised in the incubator for 30 min at 25 °C. After incubation, solution absorbance was observed at 517 nm with ethanol to avoid the reduction of some amount of antioxidant properties of AgNPs. The following formula measures the antioxidant property:

$$\mathrm{Free}\;\mathrm{radical}\;\mathrm{scavenging}\;\mathrm{rate}\;(\%)=\left[1-\left(\frac{A_{1}{-A}_{2}}{A_{0}}\right)\times 100\right]$$

Here, the absorption of solution without AgNPs is considered a negative control and subtracted during calculation, *A_0_* is the absorbance of the DPPH and filtered water at 517 nm, A_1_ is the absorbance of DPPH and BAgNPs, and A_2_ is the absorbance of ethanol and BAgNPs at 517 nm [34].

### 2.7. Statistical Analysis

The results shown here are the mean ± standard deviation. To evaluate the variances between the sets, the data were analyzed using one-way and two-way ANOVA. The data analysis was performed with the GraphPad Prism software (GraphPad, version 8.1). The significant results are shown as: *** for *p*  ≤  0.001, ** for *p*  ≤  0.01, and * for *p*  ≤  0.05.

## 3. Results and Discussion

### 3.1. Biogenic AgNPs’ Preparation and Characterization

**Figure 1:**
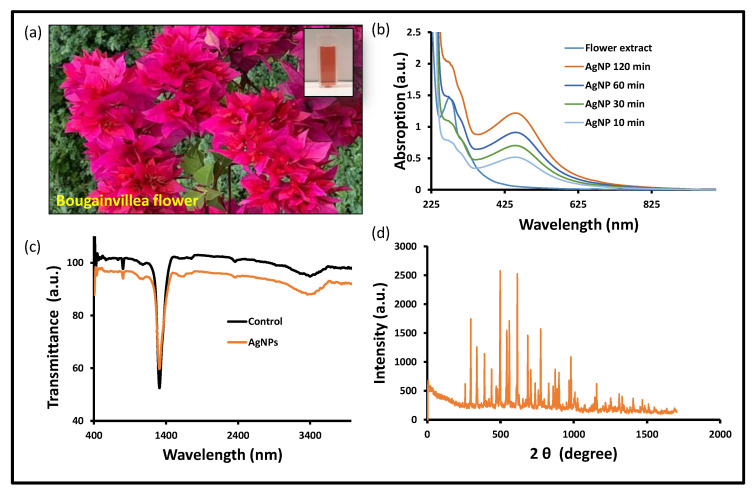
Biogenic AgNPs’ characterization: (**a**) *Bougainvillea* flower and extract in cuvette, (**b**) UV-vis spectra, (**c**) FTIR spectra, and (**d**) XRD spectra at 2 ϕ (degree) of BAgNPs.

After carefully examining the reductions of Ag^+^ into Ag^0^ in the solution, a tiny portion (1 mL) of the reaction mixture was diluted in distilled water. This reaction mixture was observed by scanning the UV-visible spectrum. During the fabrication phase, the development of a brown color solution was analyzed (Figure 1a). The reaction mixture was incubated overnight and judged by a UV-visible spectrophotometer, and a solid and sharp peak was seen at around 418–422 nm, which suggests the formation of AgNPs in the reaction mixture. In contrast, when the AgNO_3_ solution was scanned alone, no distinguishing peak was seen (Figure 1b). There have been numerous publications describing the creation of silver nanoparticles by means of green biogenic processes, and the *Bougainvillea* plant was also used for the synthesis of biogenic nanomaterials (Table 1) [31,35,36,37,38,39]. The floral extracts from the *Bougainvillaea glabra* Miami hot pink flower are still unexplored in terms of anticancer potential, including the antioxidant property and antibiofilm activity. Silver ions have been converted into their elemental form using floral extracts as reducing and stabilizing agents in an aqueous solution. Upon optimal incubation of the reaction mixture, the UV-visible absorption spectrum revealed a prominent peak between 415 and 436 nm, confirming the production of silver nanoparticles. According to previous research, the noble metal ion exhibits unique optical properties in an aqueous solution due to its distinctive surface plasmon resonance [40]. In a similar manner, during the preparation of silver nanoparticles, a color shift was observed in the reaction blend due to the reduction of silver ions (Ag^+^) into elemental silver nanoparticles (Ag^0^) during optimal incubation [41]. The nanoparticles’ size in this study is based on UV-visible spectra reported in other publications [42,43].

The biogenic nanomaterial with a biological functional group was confirmed by the FTIR study (Figure 1c). The FTIR exposed the biological functional group involved in the production of BAgNPs. The functional groups that are present were revealed by FTIR, which was used to reduce and stabilize these nanoparticles. The stretching of O-H and H- bonds represents the alcoholic and phenolic groups, and the FTIR spectra of the BAgNPs produced a similar peak at 3490–3500 cm^−1^. The intense intermediate bands, which correspond to the stretching vibrations of C=O and C=C, were also seen around 1450 and 1500 cm^−1^, respectively. Another peak, which denotes the presence of the N-H aromatic secondary amine and is attributed to the N-H stretching in the synthesized AgNPs, was observed between 1400 and 1550 cm^−1^. The *Bougainvillea* flower extract contains components such as flavonoids and terpenoids that may be held accountable for effective capping and stability for the creation of nanoparticles, as these bands correlate to bonds such as C=C and C=O. Biosynthesis of silver nanoparticles from *Cuphea procumbens* was also investigated and the biological functional group was confirmed by the FTIR [44].

The physiologically produced AgNPs’ crystalline character was revealed by XRD examination. In the spectrum of the 2 θ value, which spans 20 to 80 degrees, XRD showed four major peaks. The crystalline nature of the created materials was confirmed by the XRD peak patterns, as shown in Figure 1d. The distinct diffraction peak values were 38.15°, 44.25°, 64.40°, and 75.4° at 2θ, which indicate the reflection at (1 1 1), (2 0 0), (2 2 0), and (3 1 1) planes of the face-centered cubic structure. Additional Bragg peaks were also detected at 27.65°, 29.65°, 32.25°, 35.5°, 38.4°, 46.25°, 53.8°, and 81.05°, which were involved in silver nanocrystal formation. These additional peaks occurred due to organic compounds from the plants that stabilize and reduce silver ions into elemental nanoparticles [45].

**Figure 2:**
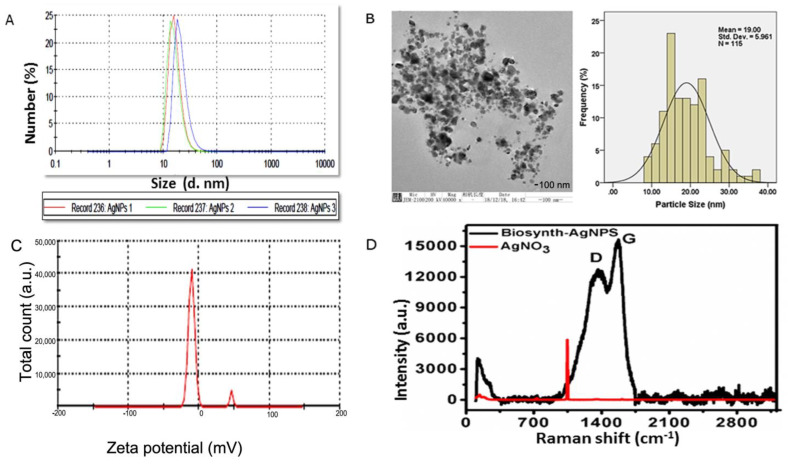
Biogenic AgNPs’ characterization: (**A**) DLS peak showing different sizes, with % values, (**B**) TEM image with distribution curve, (**C**) zeta potential of biogenic materials, and (**D**) Raman spectra of biogenic materials and control silver nitrate.

As shown in Figure 2A, DLS results showed that particle size rangedd between 20 and 100 nm, that the charge was −15.2 ± 0.5, and that the polydispersity index was 0.21. The size distribution and high negative potential support the colloidal nature and long-term stability. Consistent with the previous research, the biological synthesized AgNPs with a negative charge revealed polydispersity, in accordance with Elamawi et al.’s results [46]. Electron microscopy confirmed the results of XRD and DLS for these nanomaterials (Figure 2B). A TEM micrograph indicated that the nanoparticles produced were predominantly spherical to oval in shape. According to the TEM image, the AgNPs consisted of various particles of varying sizes in clusters ranging in size from 10 to 50 nm, and were extremely stable. Further investigation was conducted regarding the size and surface characteristics of the BAgNPs, and the spherical and oval structure of AgNPs with an average size of 19 nm was also consistent with XRD and DLS data (Figure 2C). Raman spectroscopy is a useful tool for characterizing carbon structural changes [47]. Biomaterials contain carbon derived from plant extracts, as determined by Raman spectroscopy [48]. Raman spectroscopy is an important characterization instrument to determine the disorder, defects, and carbon structure field. More recently, it is also applied to detect the presence of carbon (of plant biomaterial) as the confirmatory test for biosynthesized metallic nanoparticles. Figure 2D displays the Raman spectra of silver nitrate and biosynthesized silver nanoparticles. As biosynthesized AgNPs showed, characteristic D and G bands suggest a plant-based carbon material on the silver nanoparticles. These D and G bands were absent in the Raman spectrum of the precursor material AgNO_3_. The presence of D and G bands in AgNPs strongly suggests that their formation involved the biosynthesis process.

### 3.2. Biogenic AgNPs’ Purification and Release Kinetics

A density gradient technique was combined with centrifugal forces to purify biogenic silver nanoparticles. After incubation at 25 °C for 24 h with a reaction mixture consisting of 0.1% flower extract mixed with 0.1 molar AgNO_3_ solutions, the highest yield of silver nanoparticles, 72%, was obtained. At a 15 °C defrosting temperature in the 1 L polycarbonate flask, a parallel amount of BAgNp was recovered and appeared as a black coating. Particles in the reaction mixture froze at low temperatures, becoming denser than the medium and settling to the bottom, forming the black layer. The particles were resuspended in ultrapure water after centrifuging at 10,000× *g* for 1 h. A dialysis bag was packed and sunk into the HEPES buffer solution, where Ag^+^ ions were discharged into the medium through the dialysis membrane to study the release kinetics of biogenic AgNPs. The released ion in the medium was examined to quantify the release and the inside silver ion concentration by using an atomic absorption spectrophotometer. At the beginning of the trials, the growth of particles in the solution was lost, and only a tiny amount of silver was released. Until the reaction concluded at 24 h, there was a constant discharge of silver ions and particles into the medium from the dialysis beads (Figure S3 in Supplementary Material file). With an r^2^ > 0.98, the rate of silver dissolution followed first-order kinetics. AgNPs released into the medium are expressed as cumulative drug released percent versus time (h), with a maximum of 73.51% of AgNPs released in the medium in 24 h. The particle size and surface area impact how quickly silver ions are released. Smaller nanoparticles are discharged more rapidly than larger nanoparticles due to their great diffusibility. The biogenic nanoparticles were put in a dialysis bag and then placed in a density gradient solution; here, they released nanoparticles into the medium through the dialysis membrane. Most of the particles were released in the early stage because of the low concentration of silver, and this approach is very sensitive to detecting the initial emitted silver stage of the experiment. Throughout the investigation, there was a continuous flow of silver ions, and the rate of dissolution and concentration of silver followed zero-order kinetics according to the previously published research articles [32]. As a result, the small nanoparticles demonstrated faster release kinetics than the larger nanoparticles, most likely due to the smaller particles’ increased diffusibility [49].

### 3.3. Antibacterial Activity of As-Synthesized AgNPs

**Figure 3:**
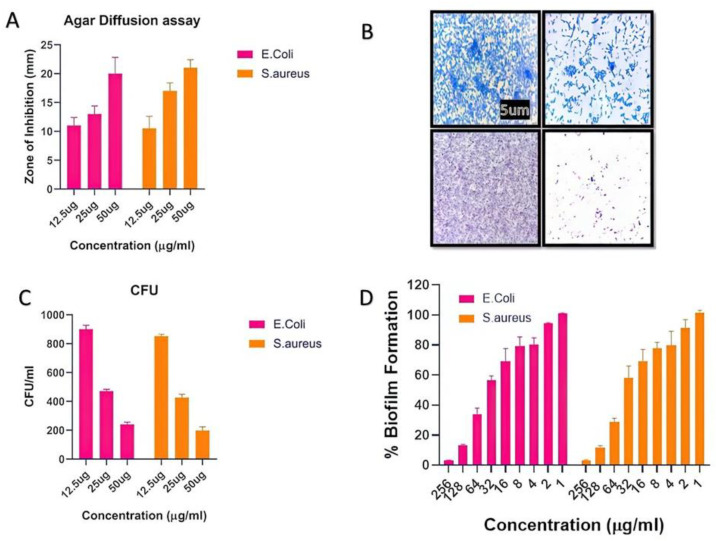
The depiction of the effect of silver nanomaterials on the bacterial growth. The (**A**) inhibition zone observed in the agar diffusion assay. (**B**) Crystal violet staining to study biofilm formation in the presence of nanomaterials, and (**C**) the colony-forming unit decreased with the increasing dose of nanomaterials. (**D**) The percent of the biofilm inhibitory effect with the increasing dose of nanomaterials.

The number of infectious diseases in society has exponentially increased, and multidrug resistance in pathogens is rising due to the abuse of antibiotics. In this regard, synthesized biogenic nanomaterials were used as antimicrobial agents against some specific pathogens. The agar diffusion assay and CFU method were used to evaluate the indirect mechanism of antibacterial action. The CFU assay results indicated that AgNPs have significant antibacterial properties. In the presence of AgNPs, there was a huge increase in the zone of inhibition as the concentration increased (Figure 3A). Further, the CFUs considerably decreased, as shown in Figure 3B. It is believed that nanoparticles possess a bactericidal effect by destroying bacterial outer membranes through reactive oxygen species (ROS), particularly hydroxyl radicals (OH), which ultimately leads to phospholipid peroxidation and bacterial death. Freshly synthesized BAgNPs have been investigated for their antibacterial activity against *E. coli* and *S. aureus* strains, which are important human pathogens. Additionally, several methods were used to evaluate the antibacterial activity of the produced silver nanoparticles in previous studies [39,42,44]. This study identified silver nanoparticles with the lowest MIC values against MRSA bacterial strains. An experiment demonstrated that biogenic green nanoparticles produced with a MIC of 10.67 ± 0.94 µg/mL against MRSA strains [50] have remarkable antibacterial efficacy [51]. We found that the MIC values for both MRSA strains were considerably lower, at 7.33 µg/mL and 6.66 µg/mL, respectively. A study conducted by Haq [52] determined that the lowest inhibitory concentrations of biogenic silver nanoparticles against *S. aureus* and *P. aeruginosa* were 100 µg/mL and 6.25 µg/mL, respectively, whereas the MIC value for *Staphylococcus aureus* and *E. coli* in the present study was 7.6 µg/mL. Furthermore, the antibacterial study was more authentic via the zone inhibition assay and found excellent results against newly synthesized BAgNPs against *E. coli* and *S. aureus* strains. The diameter of the inhibition zone (mm) test bacterial strain against AgNPs is depicted in Figure 3A. The results showed a 20 and 21 mm-diameter zone against strains of *E. coli* and *S. aureus* at a 50 µg/mL AgNPs concentration. In other studies, silver nanoparticles showed a 14 mm inhibition zone against *S. warneri* and 16 and 9.16 mm inhibition zones against *Y. ruckeri* and *L. monocytogenes*, respectively [53,54].

### 3.4. Antibiofilm Activity of As-Synthesized AgNPs

As-synthesized BAgNPs were tested for their antibiofilm properties using the XTT assay, which indicated a dose-dependent outcome on biofilm development. Due to the high hydrophobicity of their cell surfaces, bacteria can adhere to mucosal epithelial cells, phagocytes, etc. A microscopy examination revealed that both bacteria were inhibited in their production of biofilm after exposure to AgNPs. The bacterial CFU count was significantly decreased when silver nanomaterials were present in the culture media. Furthermore, the silver nanomaterial bactericidal effect was mechanistically confirmed through the damage of bacterial outer membranes by generating reactive oxygen species (ROS). AgNPs react with membrane lipid molecules, primarily generating hydroxyl radicals (OH), leading to phospholipid peroxidation and, ultimately, killing bacteria [7,32]. The high green fluorescence in the AgNP-treated cells suggested the bacterial cells’ ROS production after treatment with AgNPs, implying that the silver nano-species can physically adhere to the surface of bacteria and release an appropriate amount of ROS, eventually leading to the killing of the target bacteria. Similar ROS-generating and killing bacterial cell mechanisms were observed by several researchers [55,56].

### 3.5. Cytotoxicity Assays (MTT)

**Figure 4:**
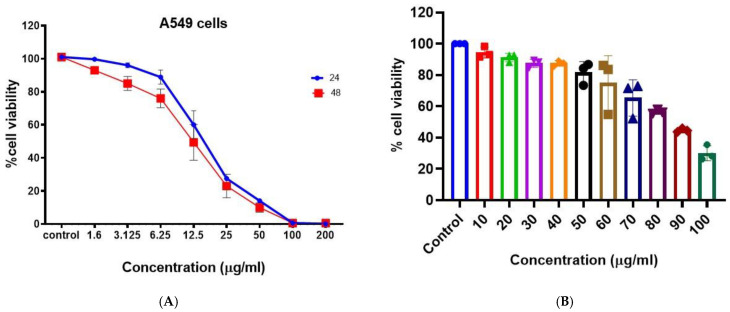
In vitro cytotoxicity of BAgNPs at different drug concentrations on the (**A**) A549 cells for 24 h and 48 h and (**B**) PBMCs for 24 h.

**Figure 5:**
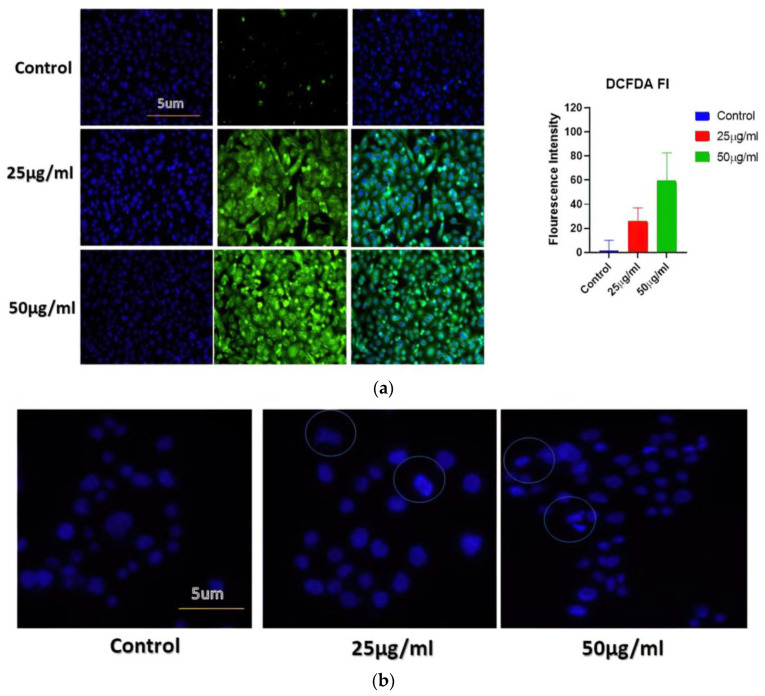
(**a**) Determination of cellular ROS generation in different cancer cell lines when exposed to increasing concentrations of BAgNPs by fluorescence microscopic analysis. Untreated cells were used as a control. (**b**) The apoptosis analysis in A549 cells using fluorescence microscopy. Here, cells were treated with BAgNPs and stained with Hoechst 322 dye and observed under the microscope.

The anticancer activity of the synthesized BAgNPs was evaluated against lung cancer cells (A549) (Figure 4A). A dose-dependent anticancer effect of AgNP was demonstrated by MTT cytotoxicity data. The anticancer effect was greater than 50% at 25 ug/mL, and the effect increased with the increasing concentration. Metallic nanoparticles, specifically AgNPs, are known to produce reactive oxygen species. The administration of AgNPs to lung cancer cells resulted in the production of ROS, such as H_2_O_2_, OH^−^, −1O_2_, etc., by the cells [44]. A variety of reactive oxygen species inhibit cellular signaling pathways and induce the death of malignant cells [57,58]. In A549 cells treated with biosynthesized AgNPs, we observed mitochondrial-dependent apoptosis, which is consistent with Gurunathan et al. [59]. In a similar study, Palaniappan et al. [60] discovered that AgNPs derived from *Cymodocea serrulata* were more potent anticancer agents since the AgNPs are fabricated in the biological scaffold and are biogenic in origin. In Figure 5a, the cytotoxicity of AgNPs is shown to be concentration- and time-dependent on A459 cells, as determined by MTT tests. It was determined that the IC_50_ value at 48 h was 17 ug/mL. PBMCs were used to assess the biocompatibility of the produced AgNPs, as indicated in Figure 4B, and biologically produced AgNPs did not exhibit significant cytotoxic results on PBMCs, even at doses of 50 ug/mL. However, as the strength of AgNPs increased, the viability of the cells decreased. This indicates the suitability of BAgNPs as a constantly stable and safe substance (Figure 4B). The use of nanoparticles in biomedicine is generally limited by the associated toxic and adverse effects [25]. As a result of their fabrication scaffolds, which contain toxic chemical agents, nanomaterials are not biocompatible. For biomedical applications, green biosynthetic techniques are highly preferred due to their biogenic origin, decreased toxicity, and adverse effects. In order to synthesize the AgNPs, we utilized extracts from *Bougainvillea* paper flowers. To determine the biocompatibility of AgNPs, we performed PBMCs cytotoxicity studies. These results indicate greater biocompatibility and decreased toxicity towards these circulatory cells, that are mostly affected by in vivo toxins.

### 3.6. AgNPs Stimulate ROS Creation in Treated Cells

The generation of ROS is studied to be one of the fundamental mechanisms by which metallic nanoparticles modify and deplete cellular proteins, DNA, and lipids, ultimately leading to cell death. DCFH-DA dye-based approaches have therefore been applied to analyze ROS produced by AgNPs (such as H_2_O_2_* anion). The DCFH-DA was oxidatively modified to yield a significantly fluorescent derivative that can be observed under a fluorescence microscope. Figure 5a shows that cells exposed to AgNPs for 24 h exhibited a significant increase in DCFH-DA fluorescence, in comparison with cells not exposed to AgNPs. Compared to untreated cells, treated A549 cells displayed higher levels of gene expression. This study indicates that BAgNPs are responsible for ROS generation, suggesting they may have an anticancer effect through the generation of ROS.

### 3.7. Apoptosis Analyzed by Hoechst Dye

The BAgNPs’ apoptotic potential was established by using the Hoechst 33342 stain. The treated cells were analyzed to judge the nuclear morphology, and we observed a homogenously stained round-shaped nuclei in control cells. Cells treated for 24 h with increasing concentrations of AgNPs (25 and 50 ug/mL) demonstrated substantial DNA condensation and the appearance of apoptotic bodies (Figure 5b).

### 3.8. Mitochondrial Potential

**Figure 6:**
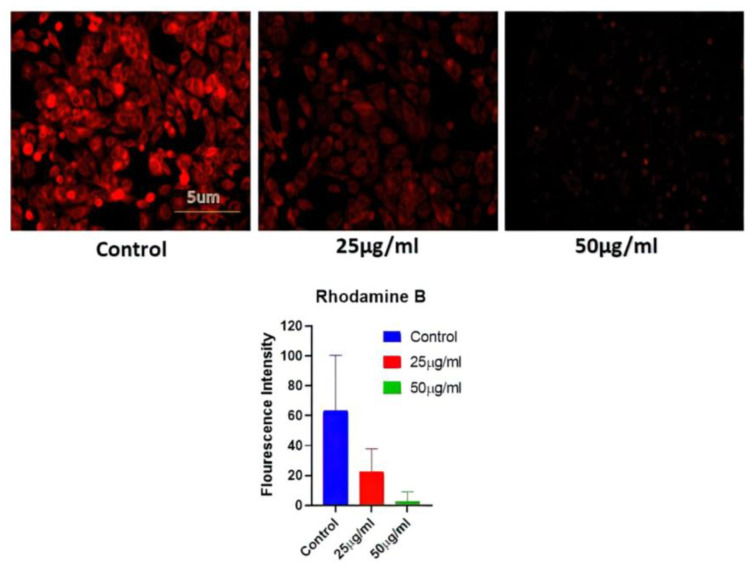
Determination of mitochondrial potential reduction after variable BAgNPs treatment in cancer cell lines by fluorescence microscopic analysis. Untreated cells were used as a control.

A549 MMP levels were measured in cells treated with AgNPs (25 and 50 ug/mL) for 24 h. Fluorescence microscopy was used to examine cell absorption of Rhodamine 231. The reduction in red fluorescence brightness was suggestive of a decrease in MMP and may be connected to the amount of apoptosis. The control group with no AgNPs treatment showed the highest level of brightness, while the AgNP-treated group exhibited a reduction in red fluorescence (Figure 6).

### 3.9. Antioxidant Activities of Biosynthesized AgNPs

**Figure 7:**
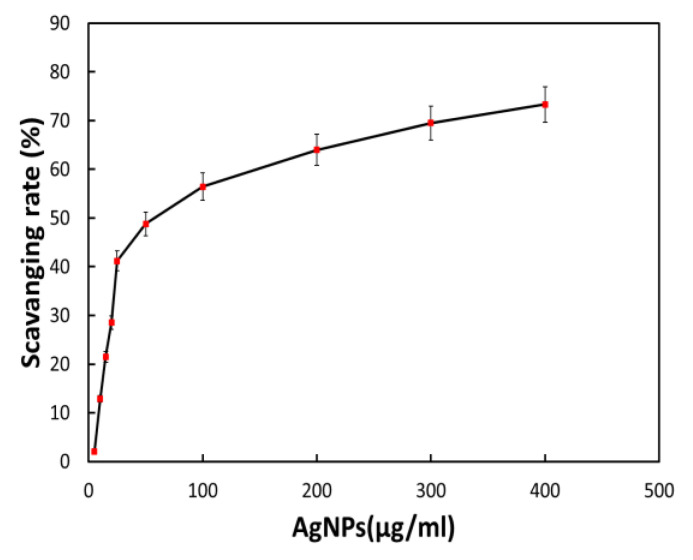
The effect of AgNPs on the DPPH assay-based scavenging under increasing concentrations.

The plant extract utilized to manufacture silver nanomaterial preparation with antioxidant capabilities contains a small quantity of plant extract or bioactive components. It was anticipated that the bioactive component of *Bougainvillea* paper flower extract, the biosynthesized AgNPs via the dried flower extract, would possess potent antioxidant effects. As a result, we assessed the antioxidant activity of biosynthesized AgNPs using the DPPH technique. The free radical scavenging rate of biosynthesized BAgNPs against DPPH solution (1,1-diphenyl-2-picrylhydrazulin) was measured, according to previous research, when AgNPs and DPPH solutions were mixed and incubated for a specific amount of time in a dark environment. When it takes hydrogen and electrons from donors, the color of DPPH varies from violet to yellow. Various amounts of biosynthesized AgNPs were added to the DPPH solution to neutralize free radicals. Figure 7 demonstrates that AgNPs had considerable DPPH scavenging activity, which increased dose-dependently and reached 41.20% at a concentration of 25.0 gmL^−1^. However, when the concentration of BAgNPs was augmented to 100 and 400 gmL^−1^, the scavenging effect was reduced to 56.44% and 73.32%, respectively (Figure 7).

## 4. Conclusions

In the present study, we established a simple green method to synthesize AgNPs using *Bougainvillea* paper flower extract. The X-ray diffraction (XRD) patterns implied that the AgNPs have a polycrystalline structure. The as-synthesized BAgNPs exhibited a significant absorption at 418 nm. The TEM and DLS patterns implied that the as-synthesized NPs have diameters between 10 and 50 nm. The FTIR and SEM analyses revealed the production of spherical AgNPs from the flower extract. The generated NPs were then evaluated for their antimicrobial and anticancer properties. The increased antibacterial activities of the produced nanoparticles were suggested by the larger zone of inhibition and the XTT assay. For the effects of BAgNPs’ therapy on the A549 lung cancer cell line, possible processes underpinning AgNPs-mediated cell death were investigated. A549 cells exposed to increasing concentrations of AgNPs exhibited lower cell viability and elevated apoptosis. One of the most significant findings of this study supported the notion that AgNPs generate toxicity in a cell-specific and proliferation-dependent way and harm the quiescent and least-susceptible rapidly proliferating cells. The substantial difference in the cytotoxic response between cancer cells and their normal counterparts offers a promising future for cancer treatment based on AgNPs. Future research should focus on in vivo studies evaluating the effects of AgNP combinations on the treatment of microbial infection and cancer management. Compared to the previously reported nanomaterials from the paper flower extract, mentioned in Table 1, the biogenic nanomaterial applied in this study is potentially more biocompatible and beneficial.

## Figures and Tables

**Table 1:** The comparative chart of *Bougainvillea* plant extract-based materials’ synthesis and biological application.

Plant	Plant Part	Material	Antibacterial	Anticancer	Size	Ref.
*Bougainvillea* flower extract	Flower	ZnONPs	+ve *E. coli*, *P. aeruginosa,* and *S. aureus*.	+ve MFC-7	40 nm	[18]
*Bougainvillea spectabilis*	Flower	AgNPs	*E. coli*	NA	10–15 nm	[19]
*Bougainvillea spectabilis*			+ve	NA	16–83 nm	[20]
*Bougainvillea spectabilis*	Flower	SeNPs	NA	NA	24–26 nm	[21]
*Bougainvillea Glabra* wild	Flower	AgNPs	+ve *E. coli and S. aurus*	NA	23 nm	[22]
*Bougainvillea Spectabilis*	Leaves and stem	AgNPs	-ve	-ve	11 nm	[23]
*Bougainvillea*	Flowers	CuONPs	+ve	-ve		[24]
*Bougainvillea glabra*	Flower	AgNPs	+ve *E. Coli* and *S. aureus,* and antibiofilm and antioxidant	+ve A549 cells and PBMCs	19 nm	In this study

Note: +ve means positive, -ve means negative, and NA means not available.

## Supplementary Materials

The following supporting information can be downloaded at: https://www.mdpi.com/article/10.3390/nano13030615/s1, Figure S1 Biogenic AgNPs optimization at different temperature when synthesized from the *Bougainvillea* flower and extract; Figure S2 Biogenic AgNPs optimization at different pH when synthesized from the *Bougainvillea* flower and extract; Figure S3. The silver nanomaterials release kinetics when it was filled in a 12 kDa limit dialysis bag submerged in HEPES buffer.
