# Supplementary material for: Therapeutic Applications of Biogenic Silver Nanomaterial Synthesized from the Paper Flower of Bougainvillea glabra (Miami, Pink)
Source: Nanomaterials (Basel). 2023 Feb 3;13(3):615. doi: 10.3390/nano13030615 (PMC9920917; doi:10.3390/nano13030615)
Supplement: Supplementary file 1 [file nanomaterials-13-00615-s001.zip › nanomaterials-2155596-supplementary.pdf]

Supplementary Materials (S.M.)

Therapeutic Applications of Biogenic Silver Nanomaterial Synthesized from the Paper Flower of *Bougainvillea glabra* (Miami, Pink)

Mohammad Oves [1,*], Mohd. Ahmar Rauf [2] and Huda A. Qari [3]

[1] Centre of Excellence in Environmental Studies, King Abdulaziz University, Jeddah, 22252, Saudi Arabia; M.O

[2] Miller School of Medicine, University of Miami, Miami, FL 33136, USA; M.A.R.; ahmarrauf2@gmail.com

[3] Biological Science Department, Faculty of Science, King Abdulaziz University, Jeddah, 22252, Saudi Arabia; H.A.Q

* Correspondence: owais.micro@gmail.com; Tel.: +966-50939957

RBC LYSIS ASSAY

In vitro erythrocyte lysis test was performed to ascertain the biocompatibility of B–AgNPs. The extent of lysis was evaluated by measuring the haemoglobin released due to membrane leakage or disruption caused by exposure the nanoparticles. Briefly, fresh blood was obtained from the hospital lab and spun at 1200 g for 10 min at 4 °C. Both buffy coat and plasma were discarded. Washed erythrocytes were diluted with isotonic buffer (20 mM PBS) to prepare 50% haematocrit. The extent of haemolysis was studied by incubating the RBC suspension with AgNPs of different concentration at 37 °C for 1 h. The incubated solutions were centrifuged at 1500g for 1 hour and the supernatant was collected and analyzed by ultraviolet-visible spectroscopy (λmax 490 nm) for released haemoglobin.

The percentage haemolysis was determined by the following equation:

$$\% \text{ Haemolysis} = [\{AbsT - AbsC\}/\{Abs(100\%) - AbsC\}] \times 100$$

where, AbsT is the absorbance of the supernatant from samples incubated with B-AgNPs AbsC is the absorbance of the supernatant from control (PBS) and Abs100% is the absorbance in the presence of 0.1% Triton X-100. Results are represented as a mean of three independent experiments.[1]

Determination of mitochondrial membrane potential in the presence of b-AgNPs

This study measured the mitochondrial membrane potential according to standard protocol. Lung cancer cells (A549) cells were revealed to B-AgNPs for a stipulated time and subsequently incubated in combination with 10 μg/ml Rhodamine-123 fluorescent dye at 37°C for 1 h in the dark. Further, two times cells were washed with PBS accompanied by analysing of the fluorescence concentration of Rhodamine-123 utilizing a fluorescence microscope (Zeiss) via capturing the images at 10X magnification.

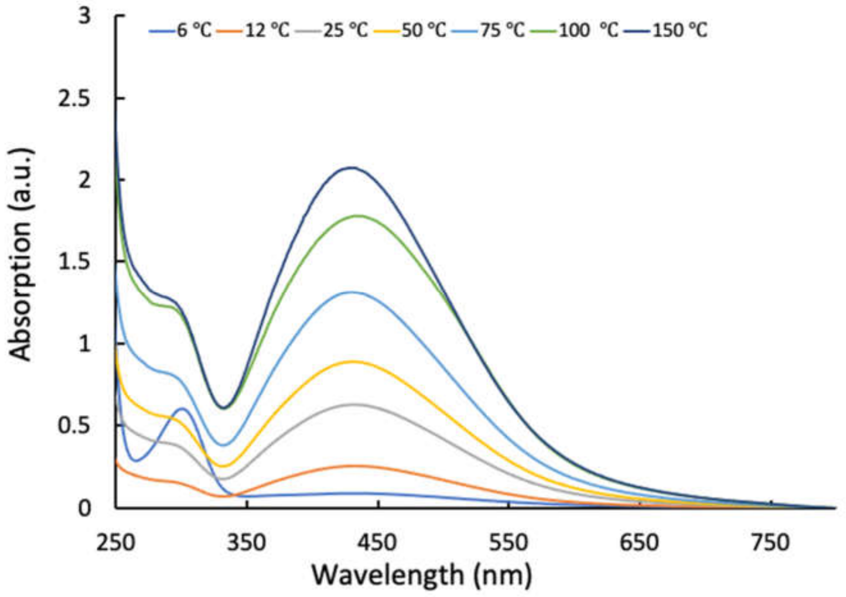

Figure S1. Biogenic AgNPs optimization at different temperature when synthesized from the *Bougainvillea* flower and extract.

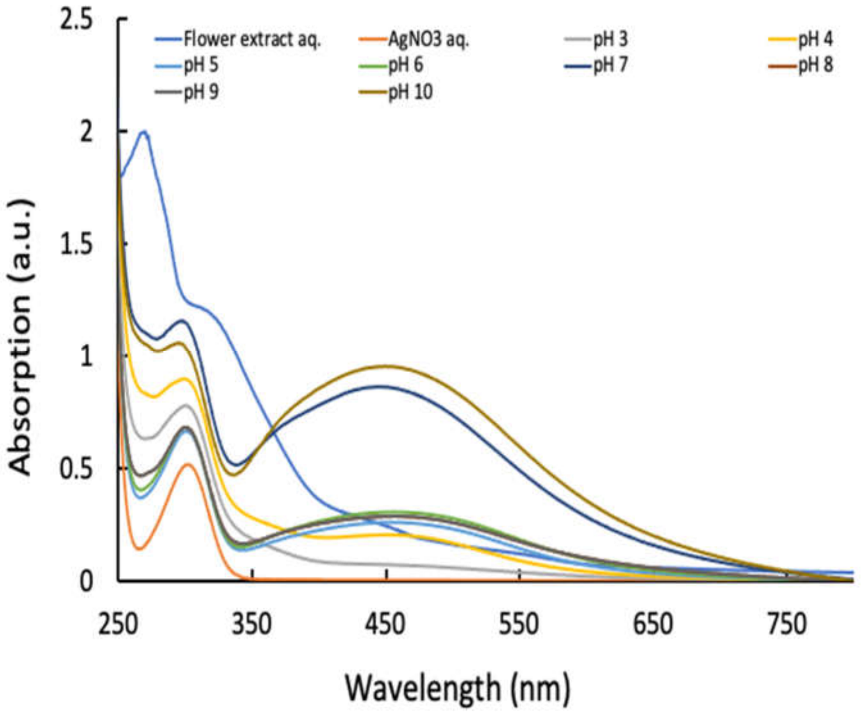

Figure S2. Biogenic AgNPs optimization at different pH when synthesized from the *Bougainvillea* flower and extract.

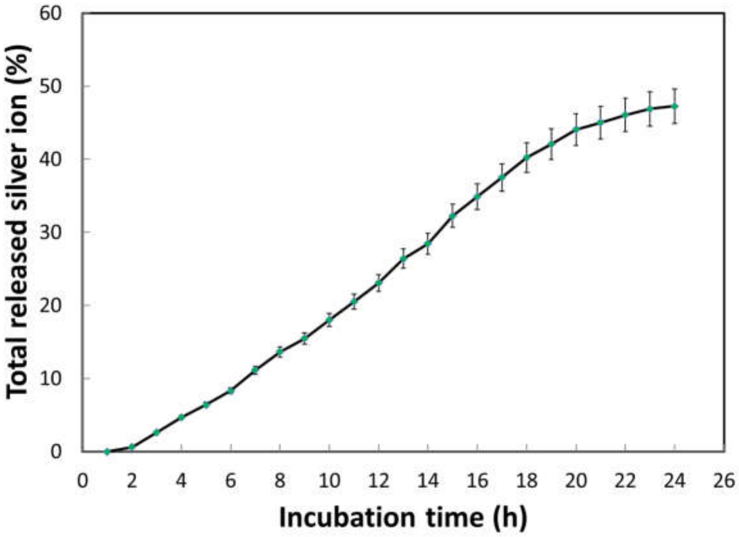

Figure S3. The silver nanomaterials release kinetics when it was filled in a 12 kDa limit dialysis bag submerged in HEPES buffer.

Reference

1. Rauf, M.A.; Oves, M.; Rehman, F.U.; Khan, A.R.; Husain, N. Bougainvillea flower extract mediated zinc oxide's nanomaterials for antimicrobial and anticancer activity. *Biomed. Pharmacother.* **2019**, *116*, 108983.
